# Exploring genetic influences underlying acute aerobic exercise effects on motor learning

**DOI:** 10.1038/s41598-017-12422-3

**Published:** 2017-09-21

**Authors:** Cameron S. Mang, Lisa M. McEwen, Julia L. MacIsaac, Nicholas J. Snow, Kristin L. Campbell, Michael S. Kobor, Colin J. D. Ross, Lara A. Boyd

**Affiliations:** 1 Hotchkiss Brain Institute, Department of Clinical Neurosciences, Cumming School of Medicine, University of Calgary, Calgary, Canada; 2 Centre for Molecular Medicine and Therapeutics, BC Children;s Hospital Research Institute, Department of Medical Genetics, Faculty of Medicine, University of British Columbia, Vancouver, Canada; 3 Department of Psychiatry, Faculty of Medicine, University of British Columbia, Vancouver, Canada; 4 Graduate Program in Rehabilitation Sciences, Faculty of Medicine, University of British Columbia, Vancouver, Canada; 5 Faculty of Pharmaceutical Sciences, University of British Columbia, Vancouver, Canada; 6 Graduate Program in Neuroscience, Faculty of Medicine, University of British Columbia, Vancouver, Canada

## Abstract

The objective of the current work was to evaluate whether the effects of acute aerobic exercise on motor learning were dependent on genetic variants impacting brain-derived neurotrophic factor (*BDNF* val66met polymorphism) and the dopamine D2 receptor (*DRD2/ANKK1* glu713lys polymorphism) in humans. A retrospective analysis was performed to determine whether these polymorphisms influence data from our two previous studies, which both demonstrated that a single bout of aerobic exercise prior to motor practice enhanced implicit motor learning. Here, our main finding was that the effect of acute aerobic exercise on motor learning was dependent on *DRD2/ANKK1* genotype. Motor learning was enhanced when aerobic exercise was performed prior to skill practice in glu/glu homozygotes, but not lys allele carriers. In contrast, the *BDNF* val66met polymorphism did not impact the exercise effect. The results suggest that the dopamine D2 receptor may be involved in acute aerobic exercise effects on motor learning. Such genetic information could inform the development of individualized aerobic exercise strategies to promote motor learning.

## Introduction

Recent work demonstrated that performance of a single bout of aerobic exercise performed in close temporal proximity to motor practice can facilitate motor skill acquisition^1–5^ and motor skill learning^3,5–7^. These behavioural findings are complemented by work demonstrating acute aerobic exercise-induced alterations in intracortical^8–10^ and interhemispheric excitability^10^, as well as increased plasticity in response to non-invasive brain stimulation protocols^3,11,12^. While the behavioural findings have potential implications for sport and rehabilitation, it is notable that there was substantial inter-individual variability in the magnitude of motor performance and learning benefits elicited by exercise in our prior work^2,3,5^. Uncovering the sources of this variability may provide insights into the mechanisms underpinning these effects and potentially inform the individualization of exercise strategies.

Past studies suggest that genetic variation contributes to inter-individual variability in neuroplasticity and motor learning in humans^13^. Considerable work has focused on a single nucleotide polymorphism (SNP) on the brain-derived neurotrophic factor (*BDNF*) gene^14–18^. This SNP (rs6265) causes a valine-to-methionine substitution at codon 66 (val66met) and is associated with impaired activity-dependent secretion of BDNF^19^, a crucial characteristic for the involvement of BDNF in long-term potentiation (LTP) mechanisms that underpin memory formation^20^. As BDNF met allele homozygotes (met/met) are relatively rare (<7% globally) (The 1000 Genomes Project Consortium, 2015), homozygous and heterozygous carriers are commonly grouped together as met allele carriers in a dominant genetic model. Previous candidate gene studies demonstrated significant associations between the presence of the *BDNF* met allele and reduced LTP-like plasticity in the primary motor cortex (M1)^14,15,21–23^. *BDNF* val66met genotype effects on motor behaviour have also been studied, but with somewhat more equivocal results. While one study suggested reduced motor performance and learning in met allele carriers^16^, others found no difference between BDNF val66met genotype on behavioural outcomes^15,22,23^. These discrepant findings have led to speculation that the effects of this SNP may interact with specific types of neuroplasticity^21^ and depend on the complexity of the skill being learned^22^. Taken together, these results also suggest that there may be other genetic factors at play in determining certain individuals’ susceptibility to beneficial effects of exercise on the motor system.

Dopamine is another key orchestrator of neuroplasticity and memory^24,25^. A dopamine-related genetic variant that may be particularly important for motor learning is located near the *DRD2* gene and within the *ANKK1* gene^26^. This SNP (rs1800497) causes a glutamic acid to lysine substitution at position 713 (glu713lys) of the *ANKK1* gene, with lys allele homozygotes (lys/lys) and heterozygotes (glu/lys) often grouped as lys allele carriers, due to a low frequency of lys/lys homozygotes in most populations (<12% globally) (The 1000 Genomes Project Consortium, 2015). Although the underlying mechanism is not well understood, human lys allele carriers exhibit reduced dopamine D2 receptor (D_2_R) availability and binding in the brain^27^. Consistent with previous work demonstrating the importance of dopaminergic signaling for motor learning^25^, a candidate gene study demonstrated that motor learning benefits elicited by administration of levo-dopa (L-dopa), a dopamine precursor, were attenuated in carriers of the *DRD2/ANKK1* lys allele compared to glu/glu homozygotes^26^.

Interestingly, animal studies demonstrated direct roles for BDNF^28^ and dopaminergic pathways^29^ in mediating aerobic exercise effects on brain function. Although neither molecule readily crosses the blood-brain barrier^30,31^, human studies reported positive relationships between increased systemic BDNF and motor learning^32^, and increased systemic BDNF and dopamine with declarative memory^33^ following acute aerobic exercise. Thus, it seems plausible that genetic variation impacting these molecules may influence acute aerobic exercise effects on motor learning. A small human study (*n* = 12) found that a 4-week aerobic exercise program enhanced declarative memory in *BDNF* val/val homozygotes and not met allele carriers, but did not consider dopaminergic gene variants^34^. Additionally, with reference to exercise effects on the motor system, past work showed that met carriers showed no significant difference in intracortical excitability after acute moderate-intensity aerobic exercise, compared to those possessing the dominant allele^9^. No prior work has considered genetic influences on acute aerobic exercise effects specifically on motor learning.

Presently, we conducted a retrospective analysis of the influence of the *BDNF* gene val66met (rs6265) and *DRD2/ANKK1* glu713lys (rs1800497) gene variants on data from our prior studies examining the effect of acute high-intensity interval cycling on motor learning^3,5^. We hypothesized that the positive effects of acute aerobic exercise on motor learning would be reduced in both *BDNF* gene met allele carriers, compared to val/val homozygotes, and *DRD2/ANKK1* glu/glu homozygotes, compared to lys allele carriers.

## Methods

### Participants

Our retrospective analysis combined data from two past studies (*n* = 16 in each)^3,5^ for a current study sample of 32 young, healthy participants with no known neurological disorders (Table 1). All participants gave written informed consent. The Clinical Research Ethics Board at the University of British Columbia approved all procedures and procedures were performed in accordance with the relevant guidelines and regulations.

**Table 1:** Summary of participant characteristics.

	All	*BDNF* val/val	*BDNF* met carrier (met/met alone)	*DRD2/ANKK1* glu/glu	*DRD2/ANKK1 lys* carrier (lys/lys alone)
Population frequency	—	62%	38% (10%)	42%	58% (12%)
N	32	14	18 (1)	17	15 (2)
CT task	16	8	8 (1)	6	10 (2)
ST task	16	6	10 (0)	11	5
Age	24.8 ± 4.2	25.9 ± 5.0	23.9 ± 3.3 (19)	24.9 ± 4.3	24.7 ± 4.3 (23 ± 2.8)
Sex	18F	8F	10F (1F)	10F	8F (1F)
Handedness	30R	14R	16R (0R)	17R	13R (2R)
VO_2peak_	44.3 ± 8.9	46.7 ± 11.9	42.5 ± 5.2 (40.2)	46.0 ± 8.1	42.4 ± 9.6 (48.6 ± 16.3)
**Ethnicity**					
White	22	11	11 (0)	11	11 (1)
East Asian	4	2	2 (1)	1	3 (1)
South Asian	4	1	3 (0)	4	0 (0)
Hispanic	2	0	2 (0)	1	1 (0)

Values presented are mean ± standard deviation. CT task and ST task refer to the continuous tracking and serial targeting tasks. Age units are in years and peak oxygen uptake (V̇O_2peak_) units are in ml/kg/min. Values in the *BDNF* met carrier and *DRD2/ANKK1* lys carrier columns represent data combining the heterozygote and homozygote carriers of the minor alleles, with values in parentheses representing only the characteristics of the met/met or lys/lys homozygotes within the larger carrier group.

### Experimental overview

**Figure 1:**
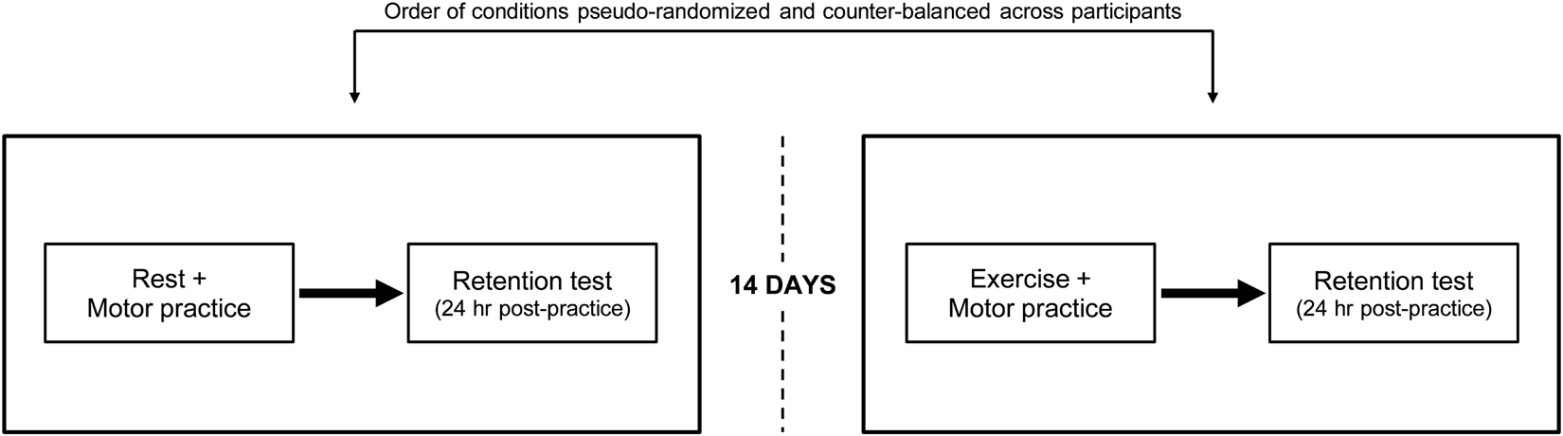
Overview of experimental procedures to test the effects of an acute bout of aerobic exercise on motor learning. A venous blood draw was collected on a separate day from all other experimental sessions. All participants completed a graded maximal exercise test prior to the experimental sessions. Order of experimental conditions (rest, exercise) was pseudorandomized and balanced across the sample.

The general experimental design was consistent between the combined studies^3,5^. The main difference between studies was the type of motor learning task employed, which is described further in the *Motor Learning Tasks* section. On a separate day from other procedures, venous blood samples were obtained from participants’ antecubital vein. Upon study completion, DNA was purified from the stored blood and the *BDNF* val66met and *DRD2/ANKK1* glu713lys polymorphisms were genotyped (custom Illumina panel, VC0013722-OPA) (Table 1 provides genotype distributions). Participants also completed a graded maximal exercise test on a separate day as described in the prior work^3,5^ to inform exercise prescription for subsequent sessions. For the experiment, a within-subjects design was employed where each participant practiced a motor task on two occasions under differing conditions. In the control condition, participants practiced the motor task immediately after resting for 20 minutes in a seated position. In the experimental condition, participants practiced the motor task immediately after completing a 20-minute bout of high-intensity interval cycling. Motor practice sessions were followed by a no-exercise delayed retention test (24 ± 2 hours) (Fig. 1). There was a minimum of two weeks between motor task practice sessions under each condition (rest or exercise)^3,5^.

### Standardized acute aerobic exercise bout

The high-intensity interval cycling bout was comprised of a 5-minute warm up at 50 W and a self-selected cadence, followed by three sets of three minutes of intense cycling at 90% of the participants’ maximal power output (in watts). These high intensity intervals were interspersed with three sets of two-minute active rest periods, in which participants maintained a cadence of at least 70 rpm and a power output of 50 W. High-intensity exercise was employed based on prior work suggesting a dose-response relationship between aerobic exercise intensity and increases in neurochemicals thought to play a role in learning processes^33^. The interval format of the exercise was chosen in an effort to minimize fatigue and dehydration, which other authors have posited could have potentially detrimental effects on cognition^35^ and motor learning^36,37^.

### Motor learning tasks

Both studies used variations of the Pew task^38^, with one task involving continuous movements and termed the continuous tracking (CT) task^3,39^, and the other requiring discrete movements and termed the serial targeting (ST) task^5,40^. Both tasks are described in more detail elsewhere^3,5^.

**Figure 2:**
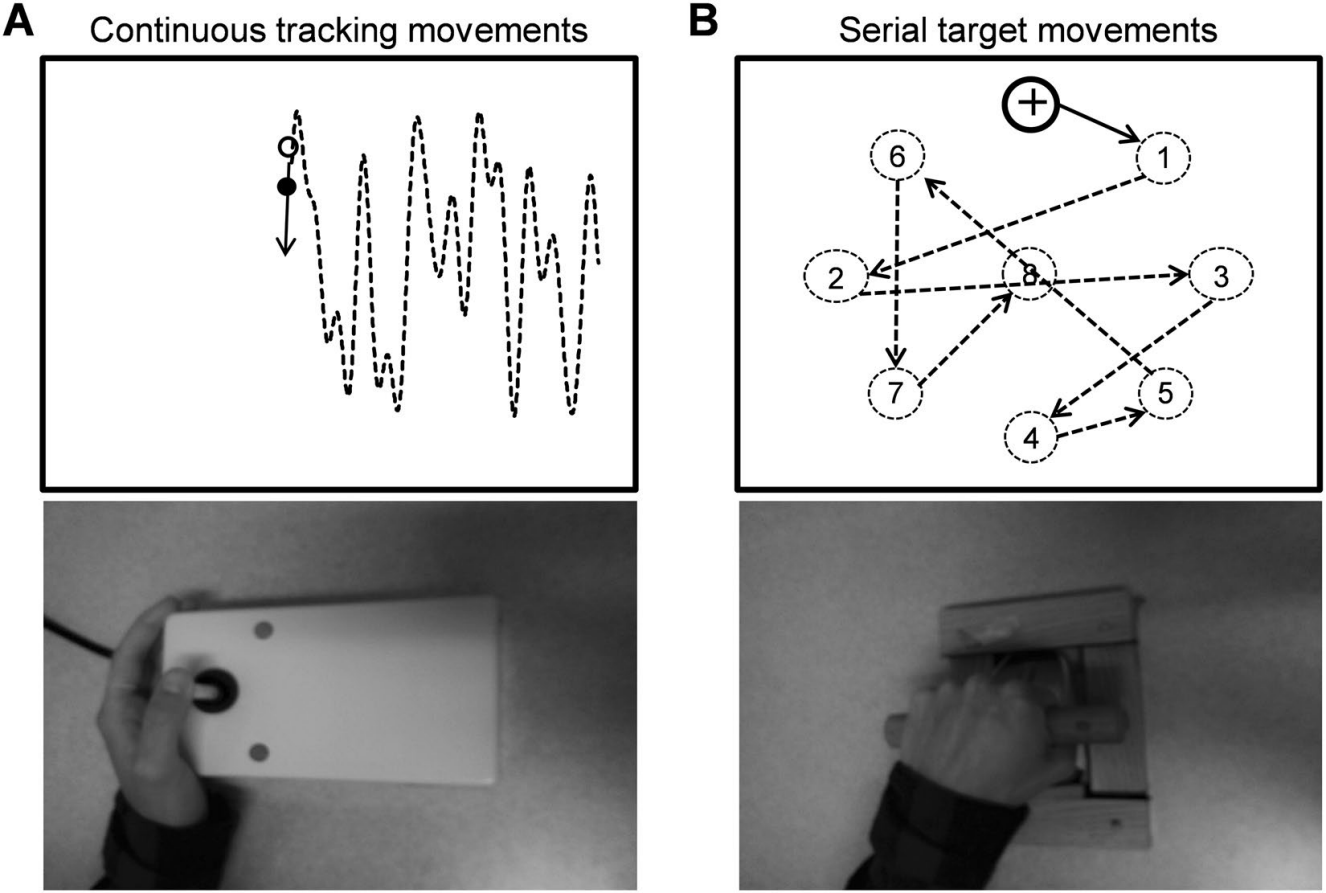
Motor tasks utilized in the two studies analyzed in the present work. Panel A depicts the continuous tracking (CT) task. The target is depicted by the black filled circle, and the cursor by the black unfilled circle. The dashed line shows an example of a target path. Panel B depicts the serial targeting (ST) task that involved performing a series of discrete movements. Targets are depicted by the black unfilled circles and the cursor by the crosshair. The arrows between targets show an example of a sequence of target movements.

Briefly, the CT task involved manipulating a small joystick with the non-dominant thumb to move a cursor to track the vertical path of a target moving at constant horizontal velocity across a screen (Fig. 2A)^3^. Participants completed a 30 s trial for task familiarization prior to the rest period or exercise bout. Motor practice consisted of two blocks of 10 × 30 s trials, for a total of 10 minutes. The following day (24 ± 2 hours after motor practice), participants completed another block of the CT task (retention test)^3^. CT task performance was quantified by the root mean square error of the participants’ cursor relative to the target movements.

The ST task involved manipulation of a computer mouse with the non-dominant hand to move a cursor between a series of targets appearing one at a time on a computer screen (Fig. 2B). At the beginning of a session, participants performed the task for 30 s for familiarization. Participants were then exposed to the rest period or the aerobic exercise bout, followed by performance of three blocks of 110 target movements, for a total of approximately 9–12 minutes of practice. The following day (24 ± 2 hours after motor practice) participants completed a single task block (retention test)^5^. ST task performance was quantified by the participants’ total response time (sum of reaction time and movement times) to complete a sequence of target movements.

Unknown to participants, in both tasks a repeated sequence of movements was practiced; this sequence was also present at the retention tests. The inclusion of the repeated sequences allows evaluation of sequence-specific implicit learning^38–41^. In the CT task, the repeated movement sequence was presented to the participant 20 times throughout the practice period (10 times per block, 2 blocks of practice). The repeated sequence in the CT task lasted 10 s in duration and was flanked by 10 s of random target movements in each 30 s trial. In the ST task, the repeated movement sequence was presented to the participant 24 times throughout the practice period (8 times per block, 3 blocks of practice). The repeated sequence was six target elements long and flanked by a random set of seven targets. For both tasks, the movement sequences were reversed between conditions, such that the sequences differed but were of equivalent difficulty for the exercise versus rest conditions. Also, the order of presentation of conditions (rest and exercise) and movement sequences (regular or reversed) were pseudorandomized and balanced across the sample for both tasks. Recognition testing following experimental sessions indicated that participants did not gain explicit knowledge of the repeated sequence, as it could not be identified at a rate higher than chance for either task (55.4 ± 16.5% and 50.0 ± 38.0% of repeated sequences correctly identified for the CT and ST task, respectively)^3,5^.

As our previous work suggested preferential effects of exercise on learning of the repeated sequence^3,5^, only data from this condition were considered in our present analyses. Measures of motor performance (root mean squared error for the CT task and response time [sum of reaction and movement times] for the ST task) were calculated for each sequence. Analyses reported in our prior work indicated no significant order effects or differences in initial performance between rest and exercise conditions^3,5^. For the CT task, performance on the first three trials of the first practice block (early practice), the last three trials of the last practice block (late practice), and the first three trials of the retention block was determined. The changes in performance from early to late practice (ACQ-∆) and from early practice to retention (RET-∆) were then calculated^3^. For the ST task, the same approach was used but with groups of four repeated sequences at each time point^5^. The slight difference in the number of trials used in the analyses for each task provided a similar proportion between tasks for the number of sequences comprising a time point to the number of sequences practiced (CT task, 3 sequences relative to 20 sequences in practice = 0.150; ST task, 4 sequences relative to 24 sequences in practice = 0.166). The datasets were combined by converting the raw change scores for each task to percent changes in performance^3,5^, where higher percent change scores indicated greater improvements. Difference scores between the exercise and rest condition were then calculated for each individual for both the ACQ-∆ and the RET-∆ variables, such that a higher difference indicated a benefit of exercise relative to rest.

### Statistical Analyses

Statistical testing used analyses of covariance (ANCOVAs). The fixed factor in all ANCOVAs was genotype (levels: SNP carrier and non-carrier), and a separate ANCOVA was run for each combination of dependent variable (difference in ACQ-∆ or RET-∆ between rest and exercise conditions) and assayed genotypes (*BDNF* val66met, *DRD2/ANKK1* glu713lys) (four ANCOVAs total). In all tests, the covariates included were participant age, sex, ethnicity, cardiorespiratory fitness (V̇O_2peak_), and the motor task used (i.e. CT and ST tasks), due to our prior work suggesting a larger effect of exercise on CT, compared to ST task, change score^3,5^. Statistical significance was set at *p* < 0.05. Parameter estimates with bootstrapped 95% confidence intervals (1000 samples) and effect sizes (η^2^_partial_) for the effects of Genotype and each covariate are reported. Effect size magnitudes were interpreted based on previously developed guidelines, such that η^2^_partial_ values below 0.06, between 0.06 and 0.14, and above 0.14 were considered small, moderate and large, respectively^42^. All statistical tests were performed using the ‘lme4’ package from R statistical software (R Development Core Team, 2008) and SPSS software (SPSS 23.0; IBM Corporation, Armonk, NY).

### Data availability

The datasets generated during and/or analysed during the current study are available from the corresponding author on reasonable request.

## Results

Results of all statistical findings are presented in Table 2.

**Table 2:** Summary of statistical results.

DV: ACQ-∆ difference	Between-subjects effects			Bootstrapped parameter estimates		
	F	*p*	η^2^_partial_	β	95% CI	*p*
BDNF val66met	1.67	0.21	0.06	7.21	−5.09, 22.57	0.28
Age	1.39	0.25	0.05	0.86	−0.92, 2.56	0.34
Sex	0.00	0.95	0.00	−0.349	−12.51, 10.78	0.95
Ethnicity	1.06	0.31	0.04	3.48	−3.75, 13.24	0.41
V̇O_2peak_	0.64	0.43	0.03	0.35	−0.38, 1.48	0.47
Task	0.33	0.57	0.01	−3.17	−16.93, 10.02	0.65
DRD2 glu713lys	1.62	0.21	0.06	−7.34	−20.79, 5.08	0.27
Age	1.27	0.27	0.05	0.81	−0.69, 2.23	0.28
Sex	0.11	0.74	0.01	−1.94	−14.29, 9.61	0.77
Ethnicity	0.94	0.34	0.04	3.23	−3.73, 12.03	0.39
V̇O_2peak_	0.07	0.79	0.00	0.12	−0.71, 0.93	0.78
Task	0.68	0.42	0.03	−4.82	−17.32, 7.93	0.47
**DV: RET-∆ difference**	**F**	***p***	**η** ^**2**^_**partial**_	**β**	**95% CI**	***p***
BDNF val66met	0.83	0.37	0.03	4.56	−6.40, 16.91	0.40
Age	0.39	0.54	0.02	0.41	−0.67, 2.11	0.57
Sex	2.14	0.16	0.08	7.60	−5.79, 18.61	0.20
Ethnicity	2.49	0.13	0.09	4.77	−2.71, 11.10	0.13
V̇O_2peak_	1.73	0.20	0.07	0.51	−0.20, 1.43	0.17
Task	0.92	0.35	0.04	−4.75	−15.73, 7.11	0.40
**DRD2 glu713lys**	**7.99**	0.01*	**0.24**	**−12.90**	**−22.47, −4.63**	0.01*
Age	1.04	0.32	0.04	0.58	−0.74, 1.66	0.28
Sex	1.79	0.17	0.07	6.08	−3.24, 15.94	0.23
Ethnicity	3.92	0.06	0.14	5.25	−0.061, 10.46	0.05
V̇O_2peak_	0.52	0.48	0.02	0.25	−0.46, 0.88	0.44
Task	3.58	0.07	0.13	−8.77	−18.52, 1.51	0.10

DV, dependent variable; asterisks indicate statistically significant (p < 0.05) results.

### Motor skill acquisition

**Figure 3:**
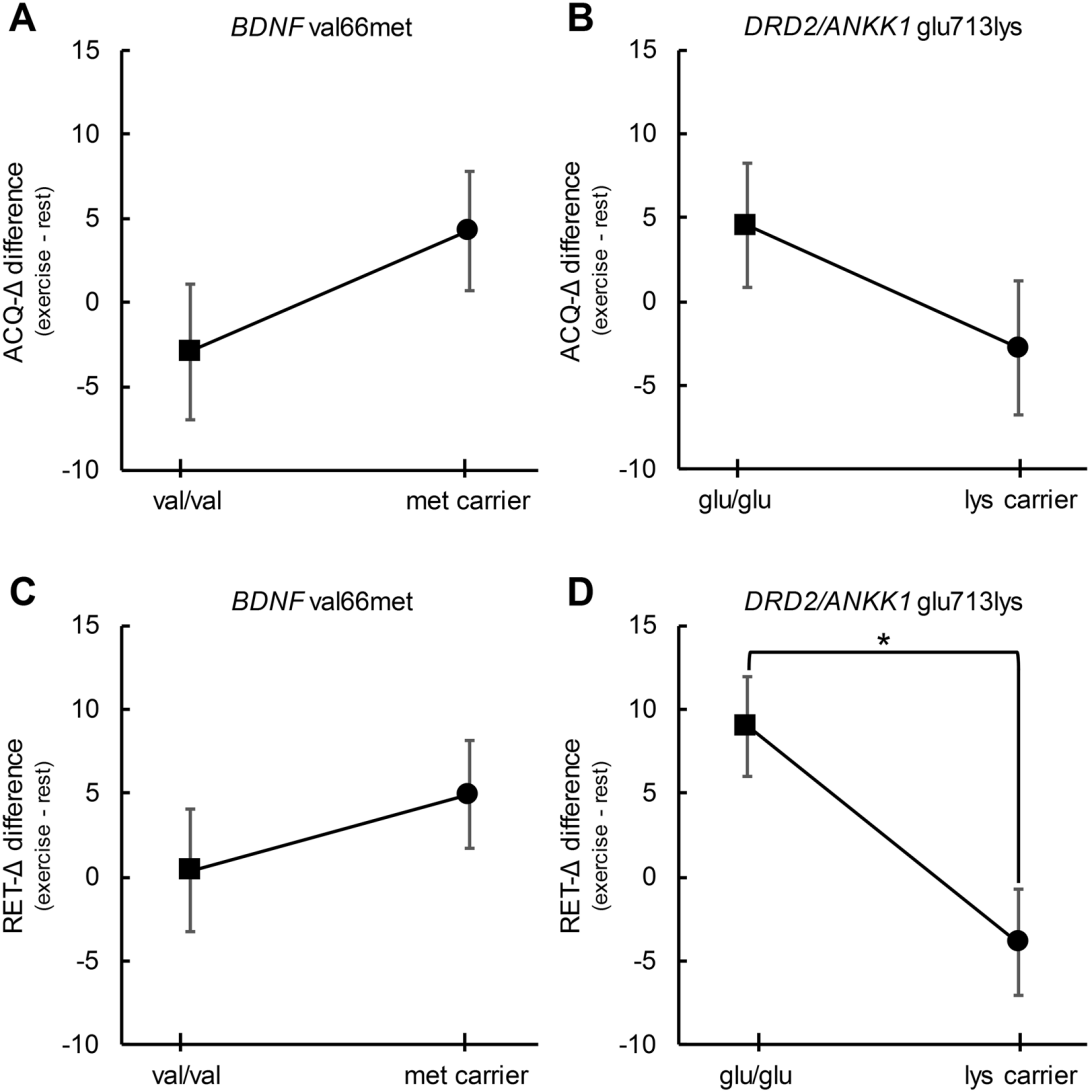
The influence of genotype on acute aerobic exercise effects on motor learning. The y-axis of all panels plots the difference between exercise and rest conditions, where a positive difference value favours the exercise condition. Panels A and B demonstrate a lack of significant difference in acquisition change score (ACQ-Δ) between genotypes for both genes of interest. Panel C demonstrates a lack of significant difference in retention change score (RET-Δ) between *BDNF* val66met genotypes. In contrast, Panel D shows a significantly different influence of exercise on *DRD2/ANKK1* glu/glu homozygotes and lys allele carriers. The means and standard error bars presented account for all covariates included in the analysis (age, sex, ethnicity, VO_2_peak and task). Asterisks indicate a statistically significant main effect of Genotype (*p* < 0.05). Values above zero indicate a benefit of exercise on motor learning.

ANCOVAs yielded no significant main effect of either *BDNF* or *DRD2/ANKK1* genotype on ACQ-∆ (*p* ≥ 0.21, η^2^_partial_ ≤ 0.06) (Fig. 3A and B). All covariates (*p* ≥ 0.25, η^2^_partial_ ≤ 0.05) and bootstrapped parameter estimates were also non-significant (*p* ≥ 0.27).

### Motor skill retention

The *BDNF* val66met SNP did not significantly influence the effect of exercise on RET-∆ (F_(1,25)_ = 0.83, *p* = 0.37) and the calculated effect size was small (η^2^_partial_ = 0.03) (Fig. 3C). Results from this ANCOVA also indicated that all covariates (*p* ≥ 0.13, η^2^_partial_ ≤ 0.09) and bootstrapped parameter estimates were also non-significant (*p* ≥ 0.13).

**Figure 4:**
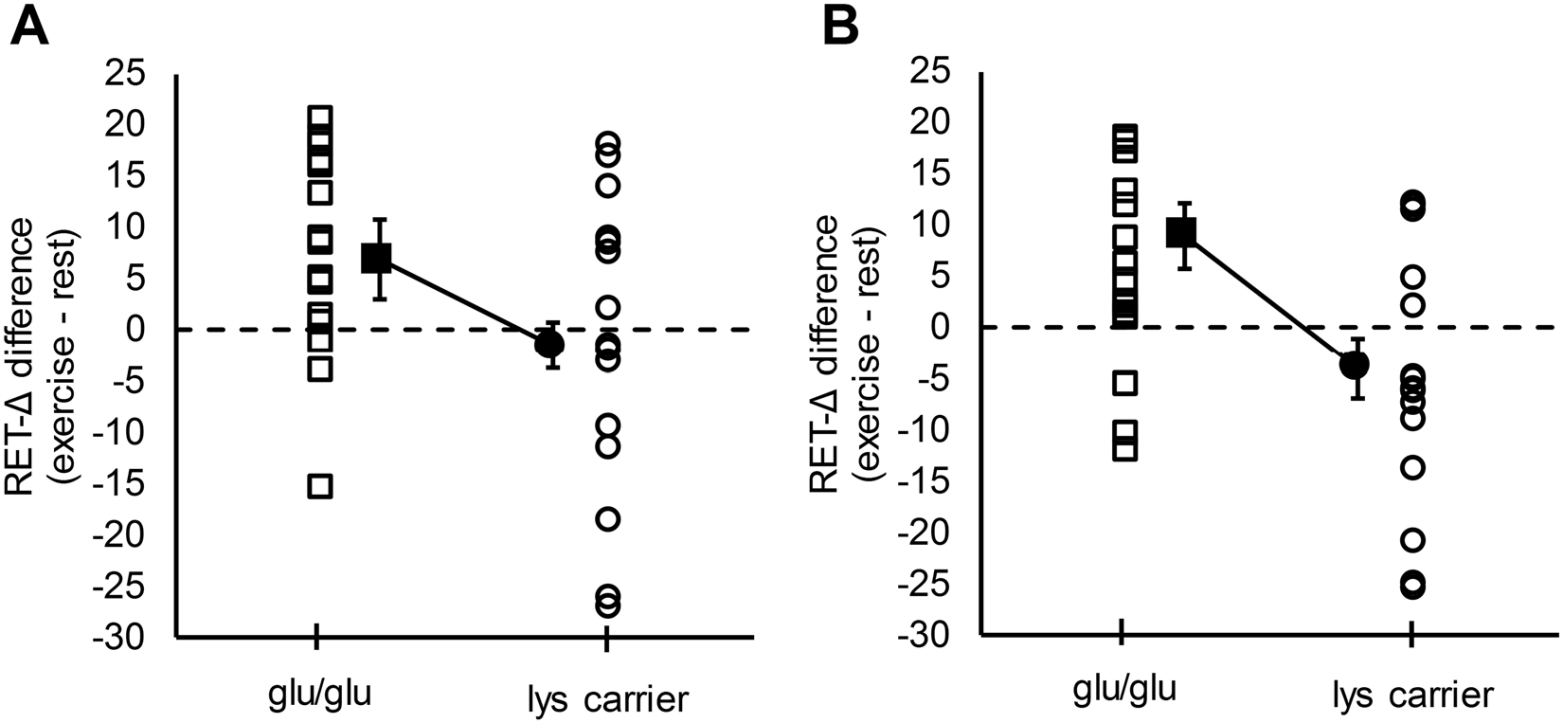
Single participant data for the significant *DRD2/ANKK1* glu713lys genotype effect on the impact of exercise on RET-Δ. Panel A demonstrates raw data from participants. Panel B demonstrates individual data corrected for the covariates included in the analysis (age, sex, ethnicity, VO2peak, task). Solid markers represents means and standard error bars for each genotype group. Values above zero indicate a benefit of exercise on motor learning.

The main finding was a significant genotype effect for the *DRD2/ANKK1* glu713lys SNP on the difference in RET-∆ between rest and exercise conditions (F_(1,25)_ = 7.99, *p* = 0.01) (Fig. 3D). The *DRD2/ANKK1* genotype effect size was large, with the ANCOVA model attributing 24% of variance in the RET-Δ difference to the absence or presence of the lys allele (η^2^_partial_ = 0.24). Moreover, the bootstrapped parameter estimate for the presence of the lys allele was also statistically significant (β = 12.90, *p* = 0.01), suggesting that the observed finding was valid for a population-level estimate. Figure 4 shows raw data (Panel A) and raw data adjusted for all covariates (Panel B) for all participants, separated by *DRD2/ANKK1* genotype. Considering raw data for each participant, 14 of the 17 (82%) glu/glu homozygotes demonstrated higher RET-Δ values under the exercise relative to the rest condition. In contrast, RET-Δ values were higher under the exercise relative to the rest condition in only 7 of the 15 (47%) lys allele carriers. Considering the covariates in the model, the Ethnicity (*p* = 0.06, η^2^_partial_ = 0.14) and Task (*p* = 0.07, η^2^_partial_ = 0.13) covariates demonstrated trends for significance with moderate effect size magnitudes.

## Discussion

Our results show that the *DRD2/ANKK1* glu713lys SNP was associated with the extent to which acute aerobic exercise facilitated motor learning, but not skill acquisition. Given that prior animal work suggests a role of dopamine in neuroplasticity and motor learning^24^ and its upregulation by aerobic exercise^43,44^, there has been prior speculation that dopaminergic signaling plays a role in acute aerobic exercise effects on memory^32,33,37^. Nevertheless, results from human studies have been inconsistent. A human positron emission tomography (PET) study indicated no change in raclopride binding to dopamine in the basal ganglia following acute aerobic exercise, with the caveat that the PET scan has low sensitivity relative to *in vivo* microdialysis studies in animals^45^. Another study found no correlation between exercise-induced increases in systemic dopamine and improvements in motor learning^32^, but acknowledged that dopamine does not cross the blood-brain barrier in appreciable amounts and that systemic levels do not necessarily reflect cortical levels^31^. However, dopamine signaling pathways in the brain involve multiple receptor subtypes (D1-D5) that each yield specific outcomes^46^. As such, exercise effects on brain function may depend on changes in specific dopamine receptors, rather than changes in levels of dopamine itself^47^. Our current findings point to a specific role for the D_2_R.

Animal and human studies of Parkinson’s disease using PET imaging suggest that changes in dopamine D_2_R expression in the basal ganglia may contribute to long-term aerobic exercise training effects on neuroplasticity and motor rehabilitation^47^. By examining a SNP known to impact expression of the D_2_R, the present study provides evidence that the D_2_R may also interact with acute aerobic exercise effects on motor learning. Notably, the *DRD2/ANKK1* genotype effect was specific to the retention change score, rather than that associated with acquisition. Previous work suggested preferential interplay between acute high-intensity aerobic exercise and consolidation of motor memory^5,36^. Likewise, dopaminergic circuits involving the D_2_R are thought to be involved in the retention phase of motor skill learning^47,48^. Taken together, these results may suggest a specific role for the *DRD2/ANKK1* SNP in moderating acute aerobic exercise effects on motor memory consolidation.

Besides work investigating acute aerobic exercise effects on motor learning, other recent studies have also examined the impact of a single bout of acute aerobic exercise on M1 intracortical excitability^8–10^. Paired-pulse TMS studies demonstrated decreased short-interval intracortical inhibition (SICI)^8,9^ and increased short-interval intracortical facilitation (SICF)^10^ immediately following acute aerobic exercise, leading to speculation that decreased γ-aminobutyric acid (GABA)-ergic inhibition post-exercise may contribute to facilitation of motor learning. A possible link between these studies and our current work lies in a human pharmacological study indicating that modulation of D_2_R binding influenced GABAergic inhibition in motor cortex; however, in this work a D_2_R agonist enhanced SICI and a D_2_R antagonist decreased SICI^49^. An alternative explanation emphasizes a role of glutamatergic neurotransmission. Acute aerobic exercise has also been shown to enhance intracortical facilitation (ICF)^9^, a paired-pulse TMS measure thought to rely on glutamatergic interneurons and possibly NMDA receptors^50,51^. Antagonism of the D_2_R receptor impairs glutamatergic-dependent synaptic potentiation and motor learning in an animal model^52^. Thus, if aerobic exercise positively impacts D_2_R expression and binding^47^, then it is plausible that the motor learning benefits observed here and in prior work are at least partly mediated by enhanced glutamatergic neurotransmission.

Interestingly, we found that motor learning was benefited by acute aerobic exercise for glu/glu homozygotes (i.e., individuals predisposed for high D_2_R expression) but not for lys allele carriers, while the opposite effect was observed for L-dopa administration^26^. Although speculative, these disparate findings may suggest differences in motor learning benefits realized from the endogenous physiological events elicited by acute aerobic exercise versus an exogenous stimulus such as administration of L-dopa. There is prior evidence to suggest that improved behavioural and neurophysiological outcomes with increased dopamine transmission follow an inverted U, such that an excess of dopamine transmission can be detrimental to behaviour and plasticity^53–57^. Indeed, the endogenous release and uptake of dopamine in response to exercise is potentially at a more inherently optimal level for the individual, as opposed to imposed, prescribed amounts. Alternatively, given differences in D_2_R expression and binding, it might be expected that glu/glu homozygotes are relatively closer to the optimal point of the inverted U than lys allele carriers when in a baseline state. The exogenous delivery of 100 mg of L-dopa in the prior work^26^ could have been an ideal stimulus for the lys allele carriers to promote motor learning, but too great of a stimulus for the glu/glu homozygotes, pushing them beyond the peak of the inverted U. In contrast, the endogenous stimulus of aerobic exercise may provide a smaller increase in the dopamine transmission that is ideal for glu/glu homozygotes, but too weak to benefit learning in lys allele carriers. If this is the case, then perhaps lys allele carriers could benefit from a greater volume of exercise than what was currently prescribed, such as an incremental exercise bout to exhaustion, which has been shown to facilitate increases in corticospinal excitability for non-exercise upper-limb muscle representations^58^. Another possibility that should also be considered is that the specific neural pathways involved in distinct memory subtypes may be influenced by the exogenous and endogenous stimuli differently and impact behaviour accordingly.

We did not detect an influence of *BDNF* genotype within our results. Given prior reports of null effects of the *BDNF* genotype on motor behavior^15,17,22,23^, this finding was not entirely surprising. Although BDNF facilitates LTP mechanisms underlying motor learning^20^ and a number of studies have shown an effect of the *BDNF* val66met SNP on M1 plasticity, motor behavior is dependent on multiple factors that may obscure detection of a genotype effect. Additionally, prior work found no impact of the *BDNF* val66met SNP on change in SICI and ICF following a bout of aerobic exercise^9^. Overall, our results suggest that, under the present experimental conditions, the *DRD2/ANKK1* glu713lys SNP may be more functionally relevant to acute aerobic exercise effects on motor learning than the *BDNF* val66met variant.

It is important to note that the genes studied in the current work are expressed across many different cell types involved in numerous biological processes. As such, inferences related to the mechanisms by which genetic variation influence the current results are speculative. Moreover, the effects of the genetic variants studied are likely dependent on complex interactions across the genome. The relatively small sample size (n* = *32) is a limitation of our study, but notably, is similar to related work (Antal *et al*.^21^, n = 29; Cheeran *et al*.^14^, n = 18; Cirillo *et al*.^22^, n = 29; Li Voti *et al*.^23^, n = 21; McHughen *et al*.^16^, n = 29; McHughen *et al*.^17^, n = 24; Pearson-Fuhrhop *et al*.^26^, n = 50). The small sample sizes in these studies and the current work are balanced by in-depth phenotyping of neurophysiological and behavioural traits. We also included a comprehensive analysis, including bootstrapping of parameter estimates and estimation of effect sizes (η^2^_partial_) to communicate the strength of our main finding as clearly as possible. Finally, the inclusion of data from two different motor tasks is another limitation of our study; however, we controlled for the type of task in our statistical analyses by including it as a covariate.

## Conclusion

In the present study, acute aerobic exercise enhanced motor learning in *DRD2/ANKK1* glu/glu homozygotes, but not lys allele carriers. The results suggest that the dopamine D_2_R may be involved in acute aerobic exercise effects on motor learning and could have implications for individualized prescription of acute aerobic exercise to promote motor learning. These findings provide an important preliminary step in elucidating the complex relationships between acute aerobic exercise, motor learning and the human genome.

## Electronic supplementary material

Supplementary Dataset 1
